# Clinical significance of type IV vascularization of laryngeal lesions according to the Ni classification

**DOI:** 10.3389/fonc.2024.1222827

**Published:** 2024-01-25

**Authors:** Lucia Staníková, Peter Kántor, Katarína Fedorová, Karol Zeleník, Pavel Komínek

**Affiliations:** ^1^ Department of Otorhinolaryngology, Head and Neck Surgery, University Hospital Ostrava, Ostrava, Czechia; ^2^ Department of Craniofacial Surgery, Faculty of Medicine, University of Ostrava, Ostrava, Czechia

**Keywords:** enhanced endoscopic methods, narrow band imaging (NBI), IMAGE1 S, Ni classification, vascular pattern, laryngeal dysplasia, laryngeal cancer

## Abstract

**Background:**

Scattered, small, dot-like intraepithelial papillary capillary loops (IPCLs) represent type IV epithelial vascularization according to “Ni classification” and are considered to be nonmalignant. According to the European Laryngological Society classification, these loops are malignant vascular changes. This contradiction has high clinical importance; therefore, clarification of the clinical significance of type IV vascularization according to the Ni classification is needed.

**Methods:**

The study was performed between June 2015 and December 2022. All recruited patients (n = 434) were symptomatic, with macroscopic laryngeal lesions (n = 674). Patients were investigated using the enhanced endoscopic methods of narrow band imaging (NBI) and the Storz Professional Image Enhancement System (IMAGE1 S). The microvascular patterns in the lesions were categorized according to Ni classification from 2011 and all lesions were examined histologically.

**Results:**

A total of 674 lesions (434 patients) were investigated using flexible NBI endoscopy and IMAGE1 S endoscopy. Type IV vascularization was recognized in 293/674 (43.5%) lesions. Among these 293 lesions, 178 (60.7%) were benign (chronic laryngitis, hyperplasia, hyperkeratosis, polyps, cysts, granulomas, Reinkeho oedema and recurrent respiratory papillomatosis); 9 (3.1%) were squamous cell carcinoma; 61 (20.8%) were mildly dysplastic, 29 (9.9%) were moderately dysplastic, 14 (4.8%) were severe dysplastic and 2 (0.7%) were carcinoma in situ. The ability to recognize histologically benign lesions in group of nonmalignant vascular pattern according to Ni (vascularization type I-IV) and distinguish them from precancers and malignancies was with accuracy 75.5%, sensitivity 54.4%, specificity 94.4%, positive predictive value 89.6% and negative predictive value 69.9%.

**Conclusion:**

Laryngeal lesions with type IV vascularization as defined by Ni present various histological findings, including precancerous and malignant lesions. Patients with type IV vascularization must be followed carefully and, in case of progression mucosal lesion microlaryngoscopy and excision are indicated.

## 1 Introduction

Laryngeal cancer is one of the most common cancers of the head and neck. Early screening and diagnosis are important for maximizing the possibility of a complete cure and to preserve the voice of the patients (1, 2). Epithelial microvascular changes could be the first sign of malignant transformation of a laryngeal lesion. Using advanced endoscopic methods — narrow band imaging (NBI) or the Storz Professional Image Enhancement System (IMAGE1 S) — and on the basis of the appearance of intraepithelial papillary capillary loops (IPCLs) in the laryngeal mucosa, we can evaluate the character of these epithelial changes and perform an “optical” biopsy (3, 4). Not only visualization, but correct interpretation and evaluation of these IPCLs is crucial for early diagnosis of precancerous changes or carcinoma.

Nowadays, various descriptive classifications of microvascular changes exist (5–8). Many scientific articles discuss the sensitivity and specificity of advanced endoscopic methods in the diagnosis of dysplastic or cancer lesions using these classifications (9–18). But there is still no direct correlation between individual classification.

**Figure 1:**
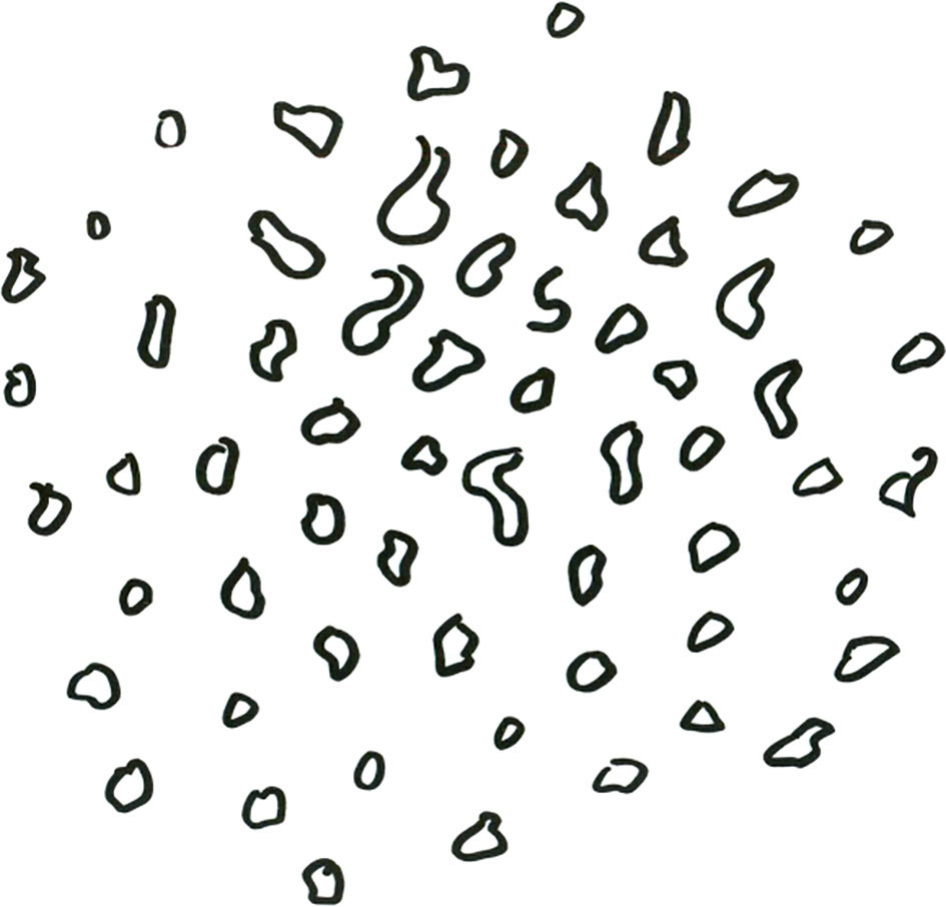
Design of type IV vascular patterns.

The evaluation of clinically and endoscopically evident benign or malignant lesions using these classifications is not complicated. However, a borderline pattern of vascularization presents scattered, small, dot-like IPCLs (**Figure 1**). Ni et al. in their original study described this pattern in 19 patients who were histologically mostly diagnosed with hyperplasia and mild dysplasia (5). Therefore, the authors considered these IPCLs classified as type IV vasscularisation to be a benign type of vascular loops. However, the number of patients enrolled was low. On the other hand, the European Laryngological Society classification published by Arens et al. describes dot-like changes as perpendicular vascularization with malignant potential (7). Based on this discrepancy represent dot-like IPCLs, IPCLs of type IV according to Ni et al. (5), a “gray” area of interpretation of vascular changes.

The aim of this study is to clarify the clinical significance of the Ni type IV pattern of vascularization in a large group of patients.

## 2 Materials and methods

This retrospective study enrolled patients examined and operated on between June 2015 and December 2022 at the Department of Otorhinolaryngology and Head and Neck Surgery of University Hospital in Ostrava. Data were collected in accordance with the Declaration of Helsinki, the requirements of good clinical practice, and all applicable regulatory requirements, and were approved by the Institutional Review Board.

All clinical records of the study population, including demographic features and information on previous treatments including laryngeal surgery, head and neck radiotherapy, or other treatments before the index transoral surgical procedure were retrieved from the hospital databases.

All recruited patients had symptoms of laryngeal disease  especially hoarseness or other voice problems. At first, outpatients were investigated for macroscopic laryngeal lesions (chronic laryngitis, leukoplakia, tumor, recurrent respiratory papillomatosis, cyst, polyp, granuloma, Reinke oedema). Enrolled patients had either a new, first-time-evaluated lesion, or a previously diagnosed chronic lesion (leukoplakia, recurrent respiratory papillomatosis, granuloma), which had changed, enlarged, and become symptomatic. Follow-up patients after laryngeal surgery and after radio- or chemoradiotherapy for cancer were also included. Included patients were without age limitation.

Patients who refused to undergo surgery with histological verification of a larygeal lesion or did not sign an informed consent to data processing were excluded from the study.

### 2.1 Outpatient NBI endoscopy

Patients were investigated using a high-definition flexible endoscope with NBI endoscopy (Olympus Visera Elite system with an Olympus OTV-S190 camera head, Olympus ENF-VH 3.9-mm rhinolaryngovideoscope, and an OLYMPUS OEV-191H monitor; Olympus Medical System, Japan) under local anesthesia in an outpatient department.

A video of the endoscopy was recorded in all cases. Microvascular changes in the lesion or in its surroundings were categorized according to the Ni classification (5). Thin or enlarged, oblique and arborescent vessels on a clear surface or under the transparent leukoplakia (type I-III vascularization according to Ni) were evaluated as benign vascular patterns. Mucosal intraepithelial papillary capillary loops (IPCLs), which are visible in a relatively regular arrangement, occur at low density, and are bifurcated or slightly dilated at the capillary terminals (type IV vascularization) are described as uncertain vascular patterns. The type IV IPCLs appear as scattered, small, dark brown spots (**Figure 1**). Brownish IPCLs various shapes, line-like or tortuous pattern with irregular distribution (type V vascularization) were evaluated as malignant vascular patterns When two or more isolated lesions were detected in one patient, each lesion was described and classified separately. Three physicians with at least a 3-year-experience in the use of NBI, blinded to the final histopathologic result, retrospectively and independently reviewed the videoendoscopic recordings.

### 2.2 Microlaryngoscopy using IMAGE1 S

Subsequently, patients underwent direct microlaryngoscopy up to 4-8 weeks after outpatient NBI endoscopy. The microvascular patterns were evaluated using the Clara + Chroma and Spectra B modalities of the Storz Professional Image Enhancement System (IMAGE1 S camera platform with a 0° or 30° rigid endoscope, Karl Storz, Tuttlingen, Germany), while ventilated via orotracheal intubation or jet ventilation in general anesthesia.

The vascular pattern visualized in Clara + Chroma and Spectra B modes were classified by the appearance. Similar to NBI endoscopy, thin or enlarged, oblique vessels were evaluated as benign pattern and irregular IPCLs of tortuous vessels were categorized as malignant vascular pattern. Scattered, small dots-like IPCLs with symmetric distribution on the surface of the lesions or in the nearest surrounding of the lesions (type IV vascularization) were categorized as uncertain vascular pattern. Three educated physicians with at least a 3-year-experience in the use of IMAGE1 S, blinded to the final histopathologic result, retrospectively and independently reviewed the intraoperative videoendoscopic recordings. Both enhanced endoscopic methods were considered to be comparable in detection vascular patterns (19). If were the vascular patterns classified differently during the NBI and IMAGE 1 S endoscopy, more serious grade of vascular changes were accepted for the next analysis. Under endoscopic control, a target biopsy from the endoscopically evaluated lesion was taken and examined histologically. In case of multiple lesions, a biopsy was taken from each lesion separately.

### 2.3 Histological examination

All laryngeal lesion of the recruited patients were histologically investigated. The lesions histopathologically defined as chronic inflammation, hyperplasia, polyp, cyst, granuloma, Reinke oedema, hyperkeratosis, and recurrent respiratory papillomatosis were considered to be benign. An invasive squamous cell carcinoma (SCC) was clearly a malignant lesion. Dysplastic changes of the epithelial layer were sorted according to the 5-grade dysplasia system (including squamous cell hyperplasia; mild, moderate and severe dysplasia; and carcinoma in situ) (20). In the study by Ni et al., the NBI vascularization was correlated with a 5-tier classification of dysplastic changes (5). In order to provide the most accurate comparison with the results of the vascularization assessment in the study of Ni et al. (5) we used the same dysplasia stratification in our study despite the existence of a new WHO classification published in 2022 (21). The diversity of histopathologic features in different groups of vascular patterns (benign/uncertain/malignant) was analyzed.

### 2.4 Statistical analysis

Categorical variables are presented as absolute and relative frequencies (%). Numerical variables are presented as medians and interquartile ranges (IQR). Between-group differences are tested using the Chi-square test of independence. The analysis of diagnostic accuracy of NBI/IMAGE1 S system was performed by the analysis of contingency tables supplemented by common diagnostic accuracy measures (accuracy, sensitivity, specificity, positive and negative predictive value). These characteristics are reported with 95% confidence intervals (obtained with the Clopper-Pearson method). Further exploratory analysis of the association of NBI/IMAGE1 S system and results of histological examination was performed using contingency tables or 100% stacked bar plot. The statistical analysis was performed using R software (version 4.3.1) and the significance level was set to 0.05.

## 3 Results

### 3.1 Clinical data

A total of 674 laryngeal lesions of 434 patients were investigated using flexible NBI endoscopy and IMAGE1 S endoscopy. The study included 305 (70.3%) males and 129 (29.7%) females, aged 2–86 years (median 60 years, IQR 46-68 years). These 434 patients had a total of 674 lesions, 484 lesions (71.8%) belonged to men, 190 lesions (28.2%) to women (**Table 1**).

**Table 1:** Distribution of laryngeal lesion in the analyzed cohort of the patients.

	Total (n = 674)	Men (n = 484)	Women (n = 190)	*P*
**NBI/IMAGE1 S**				<0.001
Benign vascular pattern	188 (27.9)	108 (22.3)	80 (42.1)	
Uncertain vascular pattern	293 (43.5)	213 (44.0)	80 (42.1)	
Malignant vascular pattern	193 (28.6)	163 (33.7)	30 (15.8)	
**Histology**				<0.001
Benign lesion	356 (52.8)	220 (45.4)	136 (71.6)	
Mild dysplasia	78 (11.6)	62 (12.8)	16 (8.4)	
Moderate dysplasia	37 (5.5)	30 (6.2)	7 (3.7)	
Severe dysplasia	30 (4.5)	26 (5.4)	4 (2.1)	
Carcinoma in situ	13 (1.9)	9 (1.9)	4 (2.1)	
Malignant lesion	160 (23.7)	137 (28.3)	23 (12.1)	

Values represent absolute and column relative frequencies (%). Frequencies are related to the number of lesions. The p-value was obtained with the Chi-square test of independence. Optical biopsy was performed by different enhanced endoscopic technique (NBI and IMAGE1 S) and more serious vascular patterns were correlated with histopathology examination. The results of the correlations are presented in the tables.

### 3.2 Analysis of vascular classification

The analysis of vascular classification according to Ni was evaluated. The benign vascular pattern (type I-III) was detected in 188/674 (27.9%) lesions. The uncertain vascular pattern (type IV vascularization) was recognized in 293/674 (43.5%) lesions. The malignant vascular pattern (type V vascularization) was recorded in 193/674 (28.6%) lesions.

### 3.3 Analysis of histological examination

**Figure 2:**
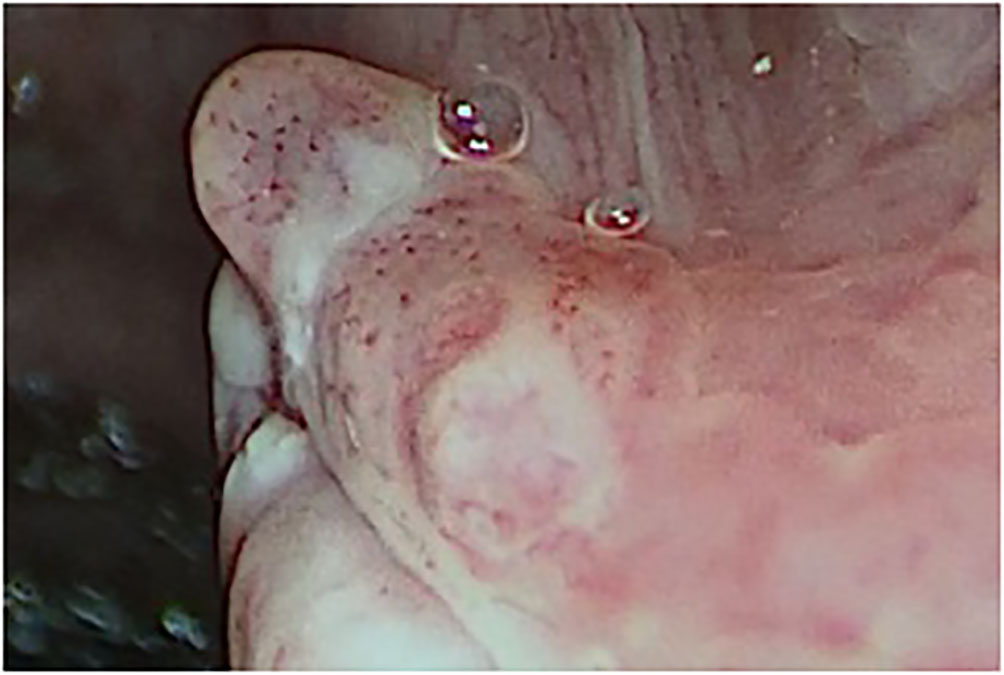
Type IV vascular pattern. Right vocal cord, benign granuloma (rigid IMAGE1 S endoscopy, Spectra B mode).

**Figure 3:**
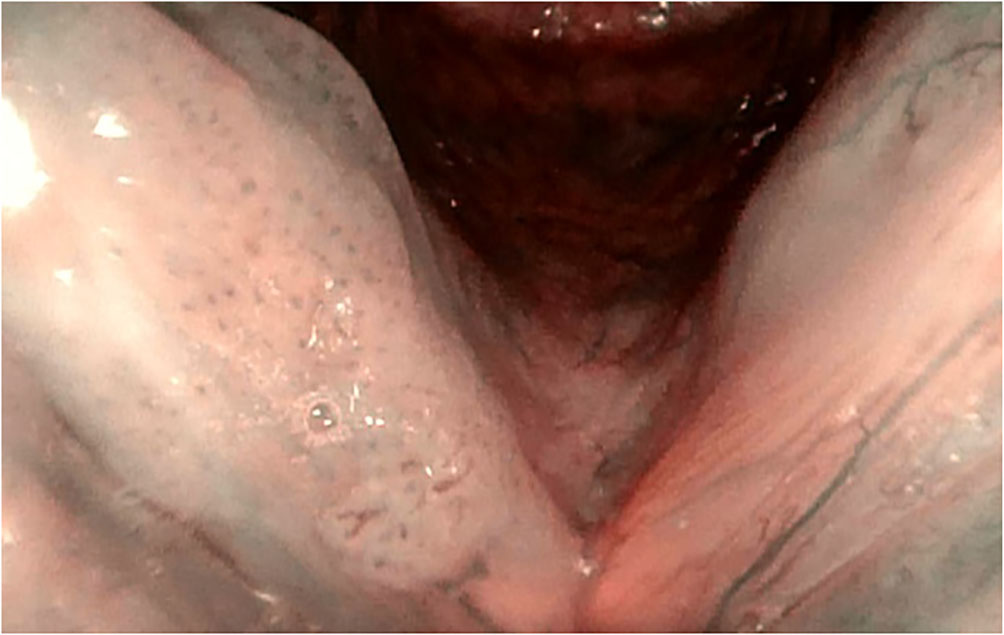
Type IV vascular patterns. Right vocal cord, recurrent respiratory papillomatosis (flexible HD NBI endoscopy).

**Figure 4:**
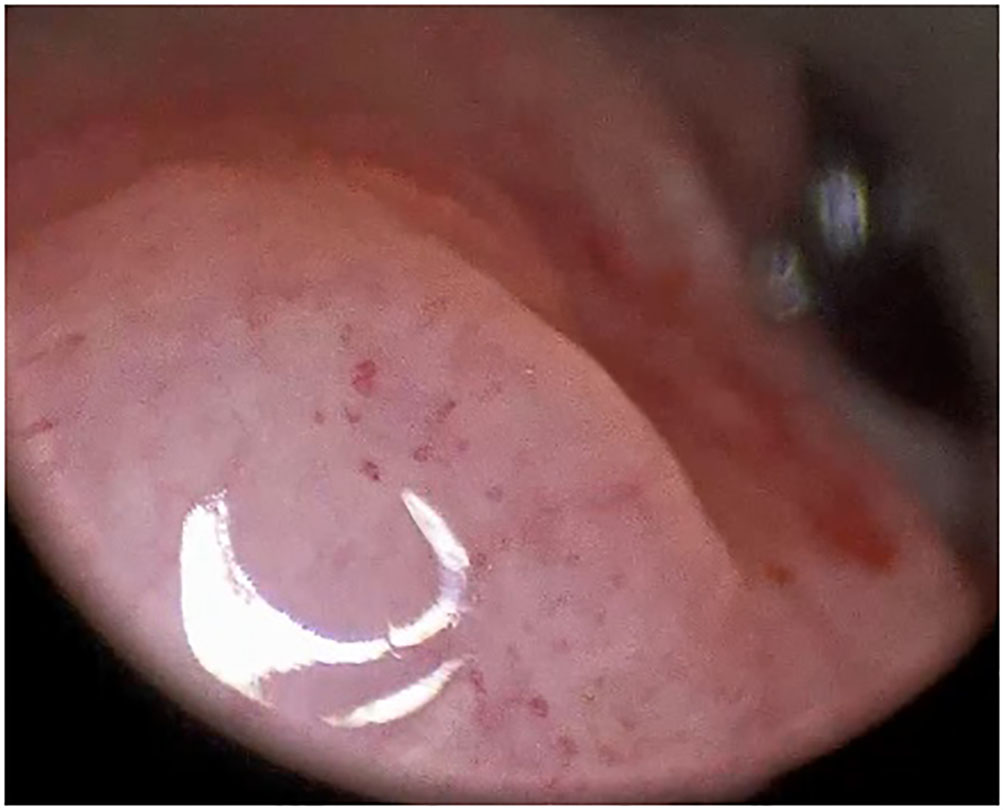
Type IV vascular pattern. Supraglottis, left arytenoid, invasive squamous cell carcinoma (rigid IMAGE1 S endoscopy, Clara + Chroma mode).

**Figure 5:**
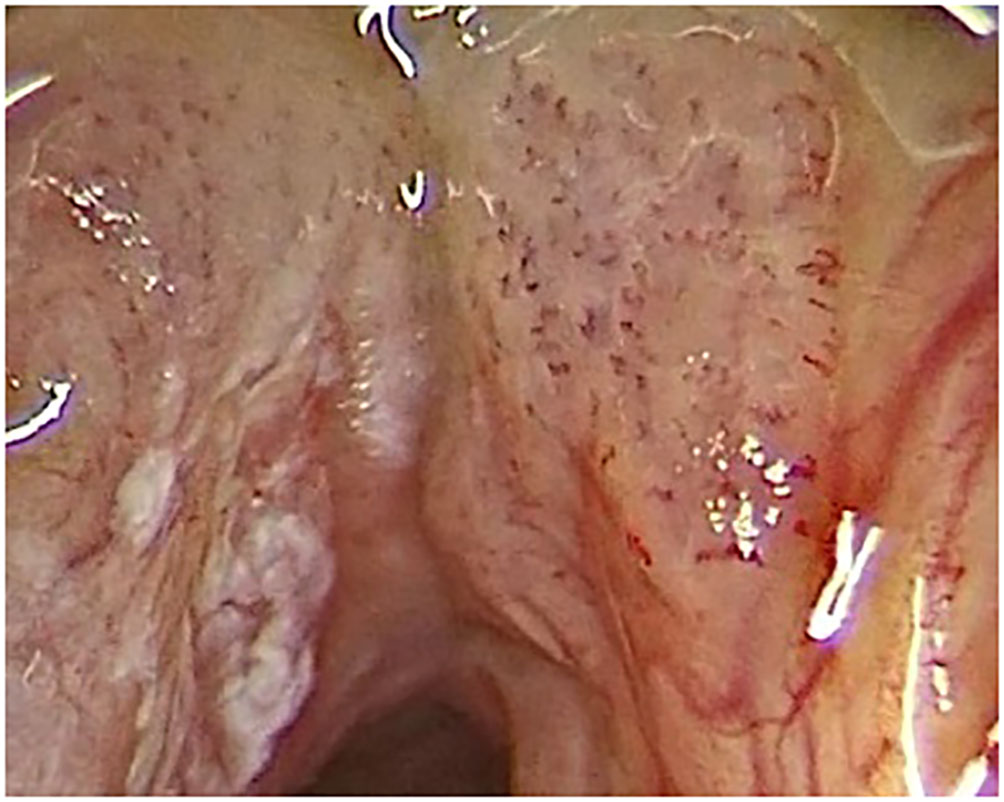
Type IV vascular pattern. Mild dysplasia on left vocal cord, moderate dysplasia on right vocal cord (rigid IMAGE1 S endoscopy, Clara + Chroma mode).

**Figure 6:**
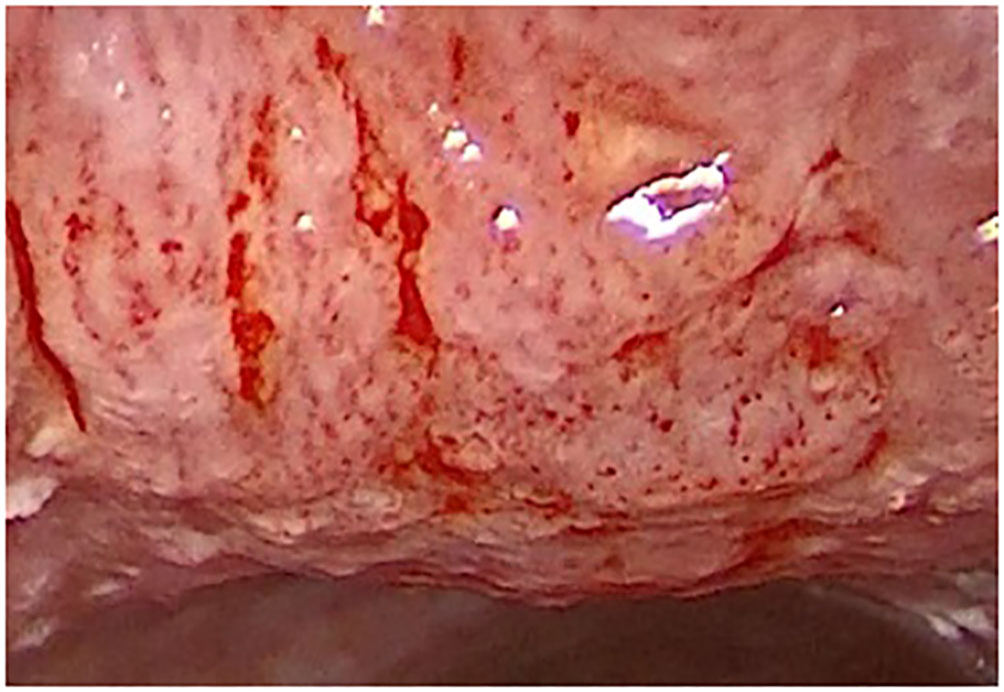
Type IV vascular pattern. Subglottis and anterior surface of trachea, severe dysplasia (rigid IMAGE1 S endoscopy, Clara + Chroma).

The histological analysis of all examined lesions was performed. Benign lesions were determined in 356/674 (52.8%) biopsies. Among those 356 biopsies, normal mucosa was detected in 14/356 (3.9%) biopsies, chronic laryngitis was recognized in 46/356 (12.9%), hyperplasia in 15/356 (4.2%), hyperkeratosis in 50/356 (14.0%), polyps in 43/356 (12.1%), cysts in 38/356 (10.7%), granulomas (**Figure 2**) in 19/356 (5.3%), Reinke oedema in 7/356 (2.0%) and recurrent respiratory papillomatosis (RRP) without any dysplastic changes (**Figure 3**) in 124/356 (34.9%) biopsies. Proportion of each benign histopathological findings is summarized in the table (**Table 2**). Malignant lesions (**Figure 4**) was identified in 160/674 (23.7%) biopsies. In the group of precancerous lesions 158/674 (23.4%), mild dysplasia was detected in 78/158 (11.6%) biopsies, moderate dysplasia (**Figure 5**) in 37/158 (5.5%) biopsies, severe dysplasia (**Figure 6**) in 30/158 (4.5%) biopsies and carcinoma *in situ* in 13/158 (1.9%) biopsies.

**Table 2:** Detailed analysis of distribution histologically benign lesions (n = 365) in correlation with vascular patterns.

	NBI/IMAGE1 S, n (%)			
	Benign vascular pattern (n = 158)	Uncertain vascular pattern (n = 178)	Malignant vascular pattern (n = 20)	Total (n = 356)
Histology – Benign lesions				
Normal mucosa	5 (3.2)	8 (4.5)	1 (5.0)	14 (3.9)
Chronic laryngitis	23 (14.6)	17 (9.6)	6 (30.0)	46 (12.9)
Hyperplasia	4 (2.5)	8 (4.5)	3 (15.0)	15 (4.2)
Hyperkeratosis	27 (17.1)	19 (10.7)	4 (20.0)	50 (14)
Polyp	38 (24.1)	4 (2.2)	1 (5.0)	43 (12.1)
Cyst	37 (23.3)	1 (0.6)	—	38 (10.7)
Granuloma	14 (8.9)	4 (2.2)	1 (5.0)	19 (5.3)
Reinke oedema	6 (3.8)	—	1 (5.0)	7 (2.0)
RRP without atypia	4 (2.5)	117 (65.7)	3 (15.0)	124 (34.9)

The values represent absolute frequencies and column relative frequencies (%).

RRP, Recurrent respiratory papillomatosis.

### 3.4 Diagnostic performance

**Figure 7:**
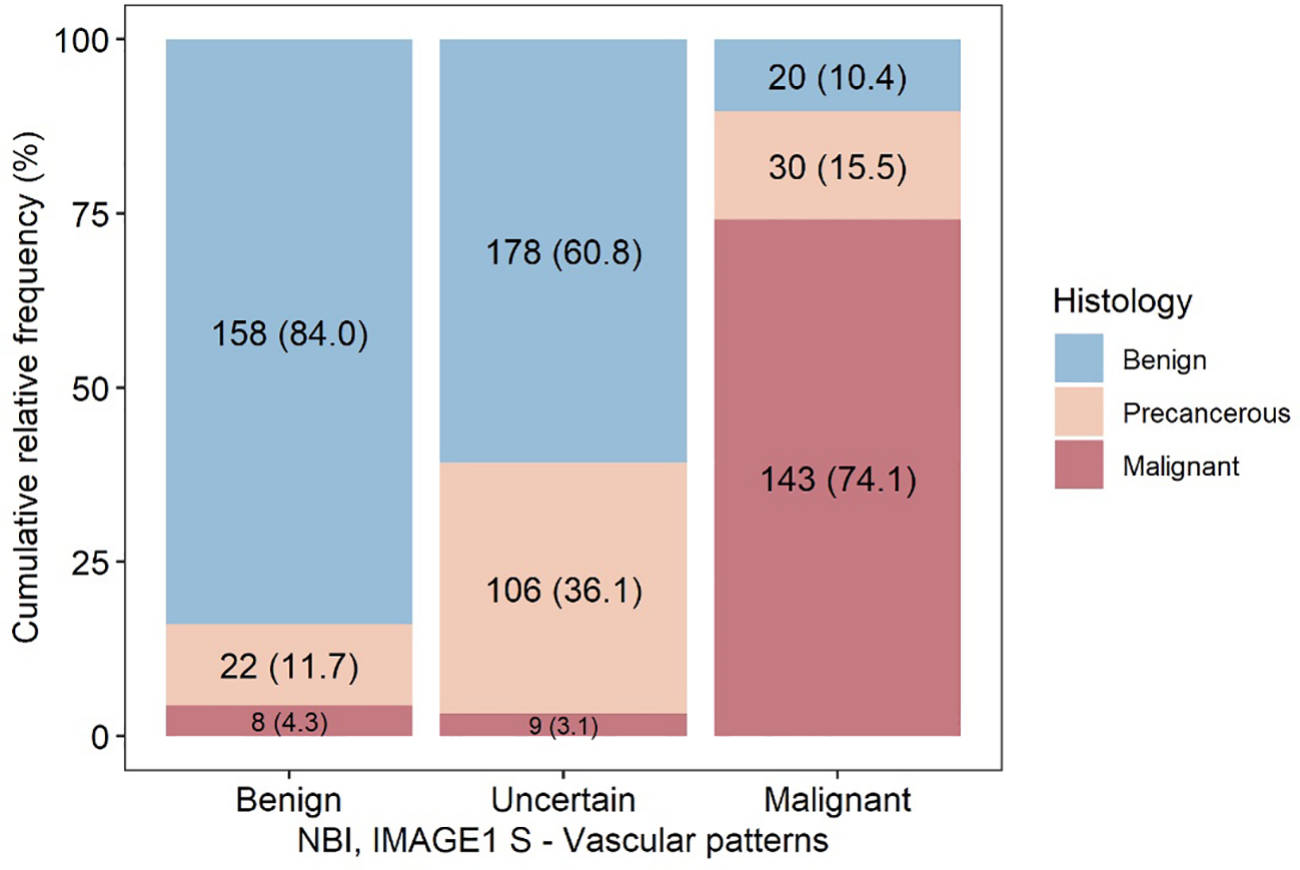
Visualization of relationship of detected vascular patterns to results of histological examination. Patients were examined by both enhanced endoscopic methods (NBI and IMAGE1 S) and more serious vascular changes were correlated with histopathology examination.

Detailed analysis of individual histological findings in relation to identified vascular patterns was performed and the resuslts are summarized in the table (**Table 3**). The absolute and relative representation of benign lesions, precancers and cancer lesions in each type of vascularization (benign/uncertain/malignant vascular patterns) is shown in the graph (**Figure 7**).

**Table 3:** Distribution of histological results (n = 674) in correlation with vascular patterns.

	NBI/IMAGE1 S, n (%)			
	Benign vascular pattern (n = 188)	Uncertain vascular pattern (n = 293)	Malignant vascular pattern (n = 193)	Total (n = 674)
Histology				
Benign	158 (84.0)	178 (60.7)	20 (10.3)	356 (52.8)
Mild dysplasia	13 (6.9)	61 (20.8)	4 (2.1)	78 (11.6)
Moderate dysplasia	3 (1.6)	29 (9.9)	5 (2.6)	37 (5.5)
Severe dysplasia	4 (2.1)	14 (4.8)	12 (6.2)	30 (4.5)
Carcinoma in situ	2 (1.1)	2 (0.7)	9 (4.7)	13 (1.9)
Malignant lesion	8 (4.3)	9 (3.1)	143 (74.1)	160 (23.7)

The values represent absolute frequencies and column relative frequencies (%).

Detection of benign vascular pattern (vascularization type I-III according to Ni) or malignant vascular pattern (vascularization type V according to Ni) using advanced endoscopic methods (NBI/IMAGE1 S) was in histological correlation with benign or malignant lesions, respectively, with diagnostic accuracy 86.9% (83.1-90.1%), sensitivity 85.2 (79.6-89.8%), specificity 88.8 (83.2-93.0%), positive predictive value 89.6% (84.4-93.6%) and negative predictive value 84.0% (78.0-89.0%) (**Table 4**).

**Table 4:** Statistical analysis of benign (type I-III vascularization according to Ni) and malignant (type V vascularization according to Ni) vascular patterns in relationship with histological confirmation of benign and precancerous or malignant lesions.

	Histology, n		Total, n
	Benign	Precancerous, Malignant	
NBI/IMAGE1 S			
Benign vascular pattern	158	30	188
Malignant vascular pattern	20	173	193
**Total**	178	203	381
Accuracy measures (95%CI)			
Accuracy	86.9 (83.1; 90.1)		
Sensitivity	85.2 (79.6; 89.8)		
Specificity	88.8 (83.2; 93.0)		
Positive predictive value	89.6 (84.4; 93.6)		
Negative predictive value	84.0 (78.0; 89.0)		

The measures are reported with 95% confidence intervals.

Vascularization type I-IV according to Ni classification is releated with nonmalignant lesions. All nonmalignant vascular patterns - benign vascular patterns (vascularization type I-III) and uncertain vascular patterns (vascularization type IV) - were detected in 481/674 (71.4%) lesions. Histological profile group of nonmalignant vascular patterns consisted of benign lesions in 336/481 (69.9%) biopsies, mild dysplasia 74/481 (15.4%), moderate dysplasia 32/481 (6.7%), severe dysplasia 18/481 (3.7%), carcinoma *in situ* 4/481 (0.8%) and invasive carcinoma in 17/481 (3.5%) biopsies. The ability to recognize benign lesions in group of type I-IV vascular patterns and distinguish them from precancers and malignancies is with accuracy 75.5% (72.1-78.7%), sensitivity 54.4% (48.8-60.0%), specificity 94.4% (91.5-96.5%), positive predictive value 89.6% (84.4-93.6%) and negative predictive value 69.9% (65.5-73.9%) (**Table 5**).

**Table 5:** Statistical analysis of non-malignant (type I-IV vascularization according to Ni) and malignant (type V vascularization according to Ni) vascular patterns in relationship with histological confirmation of benign and precancerous or malignant lesions.

	Histology, n		Total, n
	Benign	Precancerous, Malignant	
NBI/IMAGE1 S			
Benign or Uncertain vascular pattern	336	145	481
Malignant vascular pattern	20	173	193
**Total**	356	318	674
Accuracy measures (95%CI)			
Accuracy	75.5 (72.1; 78.7)		
Sensitivity	54.4 (48.8; 60.0)		
Specificity	94.4 (91.5; 96.5)		
Positive predictive value	89.6 (84.4; 93.6)		
Negative predictive value	69.9 (65.5; 73.9)		

The measures are reported with 95% confidence intervals.

**Figure 8:**
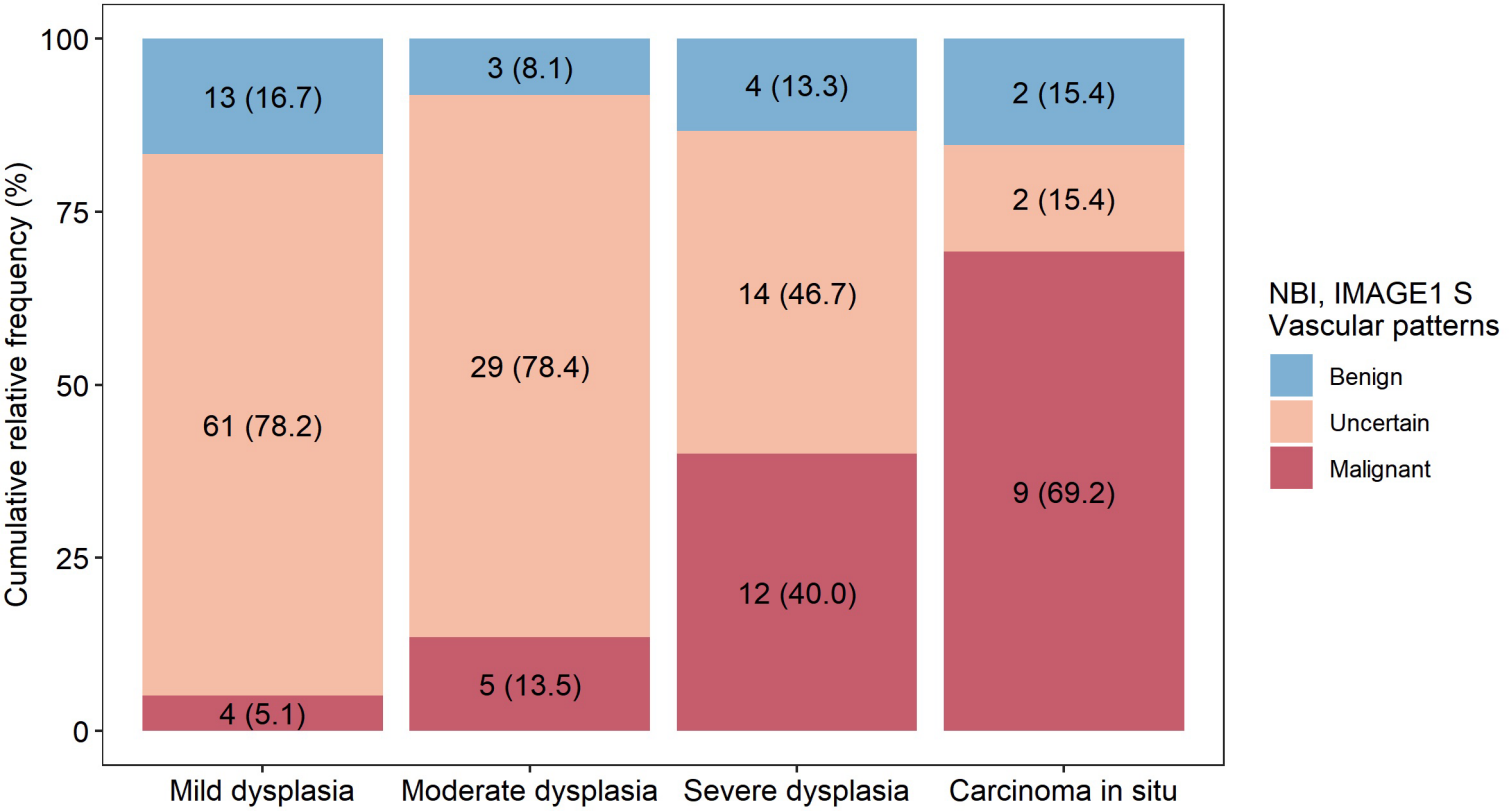
Representation of detected vascular patterns in different types of precancerous lesions. Patients were examined by both enhanced endoscopic methods (NBI and IMAGE1 S) and more serious vascular changes were correlated with histopathology examination.

In the group of uncertain vascular patterns (type IV vascularization) was detected benign lesions in 178/293 (60.7%), mild dysplasia in 61/293 (20.8%), moderate dysplasia in 29/293 (9.9%), severe dysplasia in 14/293 (4.8%), carcinoma *in situ* in 2/293 (0.7%) and invasive carcinoma in 9/293 (3.1%) biopsies (**Table 3**). The distribution of precancerous leions in relation to the assessment of vascularization is show in graph (**Figure 8**). Type IV vascularization was detected in 61/78 (78.2%) of all mild dysplasic lesions, in 29/37 (78.4%) of all moderate dysplasia, in 14/30 (46.7%) of all severe dysplasia, and in 2/13 (15.4%) of all confirmed carcinoma in situ.

### 3.5 Limitation of analysis

In the study 3 experienced laryngologists retrospectively evaluated videoendoscopic recordings of the larynx, whereas every one of them reviewed approximately one third of videos. Recordings were not analyzed by all of them and therefore interobserver reliability cannot be provided.

## 4 Discussion

Epithelial microvascular changes seem to be the first sign of structural changes, and frequently also the first sign of malignant transformation of the laryngeal lesion. Based on the appearance of intraepithelial papillary capillary loops (IPCLs) detected by NBI or IMAGE1 S, we can predict the character of epithelial changes and improve detection of laryngeal carcinoma, especially in early stages of the disease (3, 10, 22–26). Other imaging techniques, such as optical coherence tomography (OCT) and confocal laser endomicroscopy (CLE), have also shown promise for the detection of early-stage laryngeal carcinoma (27–30). The benefit of the method lies in the evaluation of the microarchitecture of the epithelium and lamina propria. OCT enhances the optical imaging of the entire thickness of epithelium and the basement membrane, which is critical in the assessment of invasiveness (28, 31). CLE imaging using fluorescein as a contrast agent allowed the visualization of the tissue architecture at a cellular level with up to x1000 magnification, resulting in additional dimension for healthy mucosa and squamous cell carcinoma differentiation contrast to NBI/IMAGE1 S endoscopy (28, 30). They can be especially helpful in the evaluation of non-transparent flat lesions, as leukoplakias or erytroplakias (28, 29), but also in assessment of the IPCLs of the normal mucosa and squamous cell carcinoma (32).

As a first Piazza et al. presented HDTV with NBI endoscopy focused on laryngeal lesions as method with high sensitivity (98%) and specificity (90%) in diagnosis malignant lesions compared to white-light endoscopy only (24). According the contemporary literature any well-demarcated brownish area with thick dark spots and/or winding vessels was judged as a “suspicious” lesion at NBI (22). Piazza et al. analyzed these “suspicious” lesions and carcinoma *in situ* or invasive carcinoma were histologically confirmed in 86.2% (24)..

The lack of adequate classification of neoplastic and non-neoplastic lesions was one of the most important flaws of existing NBI literature (33). Different levels of dysplasia being counted as “positive,” there is a lack of consistency as to what constitutes NBI positive results. Even though many studies reported a “positive” endoscopy if a well demarcated brownish area with scattered brown spots is seen, the interpretation of this in the context of an endoscopy is going to be subjective for the examiner and based on experience (34–36). There was little attempt made to account for interoperator variability.

A clear description of vascular architecture and the first classification based on the IPCL patterns in the larynx was published by Ni et al. (5). The proposed descriptive classification is probably the most well-documented classification system of these vascular changes. It has been used in numerous studies of laryngeal and pharyngeal lesions and has improved the diagnostic accuracy of NBI (3, 9, 15, 33, 37–41).

Regular oblique vessels and dendritic vessels (vascularization type I) or arborescent vessels with enlarged diameter (vascularization type II) correspond to benign lesions in 100% and 87.5–100% of cases, respectively (5, 9). On the other hand, the damaged, expanded, and distorted IPCLs with their irregularity and extension to the twisted line shape, often progressing to abnormal vessels with uneven density (vascularization type V), are characteristic of invasive carcinoma in 86.5–91.6% of cases (9, 42). Consequently, correct estimation of the histopathological diagnosis of these types of vascular features during the endoscopic examination is not problematic.

Borderline cases present visible, relatively regularly arranged, scattered, small, dark brown spots of low density, the dot-like IPCLs. IPCLs of type IV, according to Ni et al., occupy a “gray” area of interpretation of neoangiogenesis. In their original study they described this vascular pattern in 19/104 patients who were histologically mostly diagnosed with hyperplasia and mild dysplasia. The authors considered these to be IPCLs rather than a benign type of vascular loops, but the number of enrolled patients in their study was low (5). On the other hand, the European Laryngological Society (ELS) classification published by Arens et al. describes dot-like changes as perpendicular vascularization with malignant potential (7). Non-uniformity of classification, especially their views on “brown spots” or the symmetric intraepithelial capillary loops, lead to less accurate prediction of malignant transformation of these lesions in clinical praxis (11, 43).

Popek et al. analyzed 28 suspicious lesions with the type IV vascularization in the group of 333 patients with laryngeal lesions. In 17/28 cases they were classified as mild or moderate dysplasia, and 1/28 case was confirmed severe dysplasia or invasive carcinoma (40). Berlito et al. published a study of 248 patients, and the type IV lesions (12/248, 4.8%) corresponded in 100% of cases to benign and/or preneoplastic lesions (mild or moderate dysplasia) (9). Rzepakowska et al. reported a group of 91 laryngeal leukoplakia cases, which included 11/91 lesions with type IV vascularization with histological confirmation of parakeratosis or low-grade dysplasia (44). Lu et al. declared in their study of 119 patients that epithelial proliferation and low-grade (mild and moderate) intraepithelial neoplasia were mostly classified as having type III and IV vascularization (84.2%), which were significantly more common than other Ni classification subtypes. However, in the same study, 7/25 lesions with type IV vascular networks were histologically verified high-grade intraepithelial neoplasia, carcinoma in situ, or invasive carcinoma (42). Ahmadzada et al. published the results of an NBI evaluation of laryngeal cancer. They found that 10/12 cases of type IV vascularization were severe dysplasia, and 2/12 false negative cases were carcinoma *in situ* and invasive carcinoma (45). A limitation of existing studies is the number of analyzed lesions with type IV vascular patterns. Our study presents 293/674 (43.5%) lesions with type IV IPCLs, and therefore represents several times more examinations than have been reported in previously published studies. According to our results ability to recognize benign lesions in group of type I-IV vascular patterns, which are connected with non-malignant lesions, and distinguish them from precancers and cancer lesions is with low sensitivity (54.4%) and low negative predictive value (69.9%). Analysis of histopathological results in group of uncertain vascular changes (type IV vascularization) detected large portion of dysplastic changes (106/293, 36.2%) and these changes represented 106/158 (67.1%) of all dysplastic lesions in examined cohort. Low grade dysplasia was detected in 20.8% and high grade dysplasia in 15.4% lesions with type IV vessels. Type IV vascularization should be considered a marker for precancerous lesion, and thus it cannot be considered a benign or non-malignant type of vascularization.

Depending on the study, the different levels of dysplasia have been classified as “positive” or “negative,” and there is a lack of consistency as to what constitutes NBI-positive results (33, 46). It is presumed that laryngeal dysplasia has a higher rate of malignant transformation than normal epithelium (47). Van Hulst et al. specifically studied the correlation between the grade of dysplasia and development of invasive laryngeal cancer in a systematic review of the medical literature, and they concluded that rates of malignant transformation in mild dysplasia range from 0 to 41.7%, in moderate dysplasia from 0 to 48.0%, in severe dysplasia from 14.3 to 44.4%, and in carcinoma *in situ* from 11.1 to 75% (48)..

In contrast, a study of 70 patients during a 10-year period published by Luers et al. concluded that the risk of developing laryngeal squamous cell carcinoma out of laryngeal dysplasia shows no statistical correlation to the initial dysplasia grade (49). The patients with laryngeal dysplasia are an inhomogeneous group and, as stated above, the grade of laryngeal dysplasia alone seems to be an insufficient prognostic factor for the development of laryngeal cancer.

According to a recently published review of current knowledge, ELS considers the diagnosis of laryngeal dysplasia to largely rely on endoscopic procedures and histopathology. The diagnostic efficiency of endoscopy may be improved using videolaryngostroboscopy (VLS) and bioendoscopic tools such as NBI or IMAGE1 S (50). Current histological classifications are not powerful enough to clearly predict the risk to carcinoma evolution, and technical issues such as sampling error, variation in epithelial thickness, and inflammation hamper pathological examination (50). However, different studies have defined the possible utility of different biomarkers: cell proliferation factor Ki-67, cell cycle control (p 53, p16), transcription factor of embryonic stem cell pluripotency (NANOG), chromosome instability, and mutational profiles have been able to predict this risk more accurately and have demonstrated their practical utility (51–56). These methods are still in development and are not commonly used in clinical practice. Combining the assessment of IPCL patterns with molecular marker analysis might further improve the diagnostic accuracy and prognostic value of enhanced endoscopic methods.

In our study, benign lesions were detected in 178/293 (60.7%) and low-grade (mild) dysplasia in 61/293 (20.8%) of cases with type IV vascularization. In 78.2% of all mild dysplasia are these lesions represented by type IV vascularization. Especially, the high negative predictive value certifies NBI to be an excellent tool for the follow-up of certain cases of premalignant lesions with mild dysplasia, thus avoiding unnecessary biopsies or surgeries (46).

High-grade (moderate, severe and carcinoma in situ) dysplasia or SCC were confirmed in 54/293 (18.4%) of lesions categorized as having type IV vascularization in our group of patients. The risk of malignant transformation of severe dysplasia and carcinoma *in situ* to SCC is higher than in low-grade dysplastic lesions (48). This is the reason to examine the other features of the vascular network or the macroscopic appearance of the lesions to better clarify the character of vascular changes in laryngeal dysplasia.

Šifrer et al., in the study of 80 vocal cords in 40 patients, described the impact of distinguishing between narrow- and wide-angled turning points of the IPCL in differentiating between carcinoma and papilloma, respectively (43). In the other study Šifrer et al. confirmed the effectivity of ELS classification in correct interpretation of perpendicular vascular patterns with accuracy 95% (12). Lukeš et al. concluded that the most important observation for a correct diagnosis is change in IPCL shape. In distinguishing between SCC and papillomatosis, it is crucial to focus on the epithelial surface and to observe the papillary structures with a central-axis capillary and a more or less regular arrangement. Some of the associated endoscopic characteristics, such as the surface, the presence of multiple lesions, and the spread to both vocal cords, may be used as criteria for a more accurate diagnosis (25). Rzepakowska et al. presented NBI accuracy with high sensitivity and specificity (100% and 97.4%) in stratifying the risk of malignant lesions in vocal cord leukoplakia by evaluating the vessels in the epithelium surrounding the white plaque (44).

NBI endoscopy seems to be a perfect tool for the evaluation of vascular changes in the laryngeal epithelium (50, 57). However, adequate interpretation of “optical” biopsy lesions with type IV vascularization according to Ni is difficult because of the considerable variability of final histopathology results. Studies published in recent years confirm that using NBI combined with contact endoscopy to detect high-grade dysplasia and carcinoma increases diagnostic quality (13, 58).

In the future development of computer-aided diagnostic systems and investigation of computer analysis of endoscopic NBI images may further improve the quality of diagnosis (59, 60). Recent studies have demonstrated the potential of machine learning algorithms for the classification of vascular patterns in differentiating between benign and malignant lesions (59–62). Interobserver variability in the interpretation of NBI findings was one of the reason to introduce vascular classification. Study of Mehlum et al. showed that the 5-tier classification of Ni et al. (5) and Puxxed et al. (6) makes it difficult to reliably classify vascular changes in contact endoscopy and NBI even for experienced ENT surgeons. On the other hand, use of the two-level classification recommended by the ELS significantly improves the differentiation of non-cancerous lesions from cancerous lesions (63). Difficulty of clear classification the lesions is a potential limitation in the clinical application of this technique. This finding may improve the validation of IPCLs, as well as the use of artificial intelligence, might help minimize interobserver variability and improve diagnostic accuracy (60, 62).

## 5 Conclusion

Laryngeal lesions with type IV vascularization, as defined by Ni, present various histological findings, and include several precancerous and malignant lesions. Patients with type IV vascularization must be followed carefully. When mucosal lesions progress, microlaryngoscopy and excision are indicated.

## Data availability statement

The original contributions presented in the study are included in the article/supplementary material. Further inquiries can be directed to the corresponding author.

## Ethics statement

The studies involving humans were approved by Ethic Committee of University Hospital Ostrava. The studies were conducted in accordance with the local legislation and institutional requirements. Written informed consent for participation in this study was provided by the participants’ legal guardians/next of kin.

## Author contributions

LS and KZ contributed to the study’s conception and design and wrote the manuscript. LS, PKá, and KF performed the data collection and analysis. KZ and PKo supervised this study. All authors contributed to the article and approved the submitted version.
